# The relevance of music therapy in paediatric and adolescent cancer patients: a scoping review

**DOI:** 10.1080/16549716.2022.2116774

**Published:** 2022-09-29

**Authors:** Román-Carlos Rodríguez-Rodríguez, Ana Noreña-Peña, Teresa Chafer-Bixquert, Alicia Lorenzo Vásquez, Javier González de Dios, Carmen Solano Ruiz

**Affiliations:** a Nursing Department, Health Sciences Faculty, University of Alicante, Alicante, Spain; b Music and Music Therapy Department, N3 Music Centre, Alicante, Spain; c Nursing Department, University of Alicante, Alicante, Spain; d Sculpture Department, Polytechnic University of Valencia, Valencia, Spain; e Music Therapy, Pediatrics Department, Faculty of Medicine, Autonomous University of Madrid, Madrid, Spain; f Pediatrics Department, General University Hospital of Alicante, Alicante, Spain; g Department of Pediatrics, Miguel Hernández University, Alicante, Spain

**Keywords:** Music therapy, methods, oncology, paediatrics, adolescent

## Abstract

**Background:**

Music therapy is an emerging and useful methodology to improve the quality of life of children and adolescents with cancer.

**Objectives:**

The objective of this scoping review was to examine the available literature and offer an analysis of the relevance of music therapy in paediatric oncology. We considered the effects of music therapy on children and adolescents with cancer as well as the perception of this population, their families, music therapists, and health professionals regarding the music therapy sessions conducted. Finally, we analysed the characteristics of the distinct types of music therapy interventions reported in the literature.

**Methods:**

In this review, we applied the methodology proposed by Arksey and O’Malley. After performing a comprehensive academic literature database search, 522 articles were identified of which, 27 met the inclusion criteria.

**Results:**

The results shed light on the use of music therapy as a means to facilitate self-esteem, to improve the physical, emotional, and cognitive aspects related to disease and, to a lesser extent, alleviate their physiological symptoms. Both children and adolescents with cancer were represented in the academic literature. The most prevalent findings described in these studies were the benefits of music therapy in terms of improved psychological well-being and social relationships in this population.

**Conclusions:**

Music therapy interventions are generally well received, not only by children and adolescents with cancer, but also by their families, music therapists, and health professionals. Nevertheless, several gaps were identified in some of the studies we considered, including a lack of specificity regarding the results obtained or music therapy intervention methods used.

## Background

The World Health Organisation estimates that worldwide, about 400,000 children and adolescents under the age of 19 are diagnosed with cancer each year [1]. The most common cancers in this population are leukaemia, lymphoma, brain tumours, solid tumours, and Wilms tumour [2]. Apart from suffering with these diseases, these children must live through very disturbing and stressful life situations resulting from the numerous invasive tests they must undergo and the aggressive treatments they receive. This often means they spend a lot of time in unfamiliar surroundings and, moreover, completely upends their daily routines. This complete lifestyle change separates them from their friends, school, routine activities, and reduces contact with most of their extended family. This situation also creates physical and emotional problems for their parents or caretakers. Thus, the consequences of all of the above tend to negatively influence the mood and quality of life of patients and their families [3,4].

There is a growing interest in the use of integrative medical techniques, such as, but not limited to, music therapy (MT), acupuncture, and massage. These treatments aim to palliate the psychosocial symptoms associated with the diseases they suffer from and their treatments and is aimed at both patients and their families [5–7]. MT is an effective in-hospital treatment that is being applied in all areas of medicine and in every age group. Its use has been studied in the fields of cardiology [8,9], neurology [10–12], psychiatry [13], and intensive care [14,15], among others. MT has been used in oncology in patients of all ages since the 1970s and has been shown to be a powerful tool to improve patient quality of life [16].

Music therapists stress the importance of using live musicians to perform songs [17] because this promotes interaction among family members, allowing them to express themselves with greater freedom [18]. Studies on the use of MT in paediatric cancer patients, some of which had terminal disease, first appeared in the 1980s. The purpose of these studies was to help children channel their feelings of fear and manage their anxiety [19]. Other studies have dealt with the physical, psychological, emotional, and social needs of patients [20,21], and stressed the special relevance of studies involving qualified music therapists [22].

The American Music Therapy Association defines MT as ‘The clinical and evidence-based use of music interventions to accomplish individualized goals within a therapeutic relationship by a credentialed professional who has completed an approved music therapy program. Music therapy interventions can address a variety of healthcare and educational goals: promote wellness, manage stress, alleviate pain, express feelings and more’ [23]. This definition emphasises the importance and value of MT in patients with long-standing medical problems – such as paediatric oncological patients – which leads to physical, psychological, and emotional problems [24]. MT uses theoretical models that include behavioural, psychoanalytic, and humanistic orientations implemented using several different methods. In active MT methods, the patients sing, play instruments, and improvise, while receptive MT methods have the patients listen to recorded or live music. Finally, mixed methods combine both these elements [25].

Research on MT in paediatric patients using a variety of methods and techniques has demonstrated positive results [26–30]. Studies, such as that of Orrigo [31], have shown that MT has physical and psychological benefits in paediatric cancer patients, including self-expression, providing a useful distraction, and improving relationships with others. MT interventions can also help the families of paediatric oncology patients [30,32]. Indeed, recent systematic reviews on the use of MT with children and adolescents with cancer [33–35] have shown that most studies have had positive effects on physiological, psychological, social, and physical parameters in these patients, which in turn has improved their well-being. However, these reviews also refer to the heterogeneity and flaws in the study designs of the MT interventions completed to date.

Considering the above, the objective of this scoping review was to examine the available academic literature and analyse the relevance of MT in paediatric oncology. We considered the effects of MT on children and adolescents with cancer as well as the perception of this population, their families, music therapists, and health professionals regarding the music therapy sessions conducted. Finally, we analysed the characteristics of the distinct types of MT interventions reported in the literature.

## Methods

### Scoping reviews

Scoping reviews are a type of research synthesis that aims to map the literature on a particular topic or research area and provide an opportunity to identify key concepts, knowledge gaps, and types and sources of evidence that can inform policymaking and practice guidelines, and so on [36]. They allow researchers to identify the degree of development in a particular field, and can be particularly useful when evidence related to a selected topic is still emerging [37]. Given the emerging evidence on the use of MT interventions with paediatric cancer patients, we decided that a scoping review would be the most appropriate approach to identify how MT has been used in this population to date. Our aim was to inform readers about important developments in this arena that are becoming more prominent in the scientific literature, even though this previous work had often reported heterogeneous results. Nonetheless, in our opinion, these studies demonstrate the current academic perceptions of MT and discuss optimisation of their design.

This scoping review was designed using the methodology proposed by Arksey and O’Malley [38]. Briefly, they recommended not establishing strict limitations on the search terms at the beginning of the work in order to identify all the possible relevant studies. Thus, this process is not linear but rather, is iterative. This requires researchers to be thoughtfully engaged with each stage of the review process and, if necessary, to repeat steps to ensure that the literature is comprehensively covered. This methodology comprises six stages, with the sixth being optional. In the current work, we chose to incorporate the first five stages: (1) identifying research questions; (2) identifying relevant studies; (3) study selection; (4) charting the data; and (5) collating, summarising, and reporting the results. Furthermore, the PRISMA Extension for Scoping Reviews (PRISMA-ScR) criteria [39] were used as guidelines when reporting our findings.

#### Stage 1: identifying research questions

Our objective was to examine the available literature and offer an analysis of the relevance of MT in paediatric oncology. To better understand this topic, we considered the following: (a) the effects of MT in children and adolescents with cancer; (b) the perceptions of children and adolescents as well as their families, music therapists, and health professionals regarding MT sessions; and (c) the characteristics of MT interventions. Our intent was to explore the following research questions:

- What types of evidence exist regarding the treatment of children and adolescents with cancer by using MT?
- What are the effects of MT as applied to children and adolescents with cancer?
- How do children and adolescents with cancer, their families, health professionals, and music therapists feel about MT interventions?
- What are the main features of the current MT interventions being used with children and adolescents with cancer?

#### Stage 2: identifying relevant studies

To identify relevant studies, we developed a key inclusion criteria based on the population–concept–context (PCC) framework, as recommended by the Joanna Briggs Institute for scoping reviews [40] (see Table 1 for the study inclusion criteria). We then developed a method for identifying relevant studies using a three-step literature search strategy that balanced the viability, breadth, and comprehensiveness of the studies, as recommended by Khalil et al. [41]. The first step was a limited search to test the selected keywords using Ovid MEDLINE; the second step used index terms and all the keywords identified using all the search databases employed (i.e. PubMed, CINHAL, PsycINFO, Dialnet, and SCOPUS); and the third step was an analysis of the reference lists provided in the studies identified for consideration.

**Table 1.** The population–concept–context framework.

PCC	Inclusion criteria
Population	Children and adolescents with cancer.
Concept	Research studies of music therapy interventions with children and adolescents with cancer to promote their health and enhance their quality of life, available in English and Spanish between January 2002 and July 2021.
Context	Qualitative and quantitative research analysis and studies that combined both methodologies.

PCC = population–concept–context.

Given the scarcity of publications on MT in paediatric oncology, we decided to retrospectively extend the search until the year 2000 in order to improve the quality of the review and to add to relevant articles on the subject. The comprehensive database search was conducted between 9 March 2020, and 10 November 2020, at two separate times using different databases. Between 9 March and 30 May 2020, a preliminary search was carried out using the article titles found in the PubMed, CINHAL, and PsycINFO databases. In turn, between 1 June and 31 July 2020, a second search was performed in the same databases, which included the abstract in the search field. An identical approach was later used for the Dialnet and SCOPUS databases where we conducted a preliminary search focused on the article titles between 1 August and 30 September 2020, with the second search that included the abstract being carried out between 1 October and 10 November 2020.

The search was updated between 1 February and 1 August 2021. The keywords were obtained from health sciences descriptors thesaurus (DeCS in its Spanish initialism) which encompasses Portuguese, Spanish, and English search hits as follows: music therapy, paediatrics, and adolescent. In addition, we also searched for the following words in Spanish or English: cancer, tumour, neoplasia, oncology, and children. These terms were combined using the Boolean AND/OR operators as well as some truncated operators. Filters were applied to retrieve only publications written in English or Spanish between January 2002 and July 2021 so as to include as many articles as possible in the review. Following the database search, the reference lists from the identified studies were also analysed.

#### Stage 3. study selection

The initial search identified 522 articles. Screening of the results by title and abstract was performed independently by the first two authors of this manuscript, R.R. and A.N. Duplicates and irrelevant records (e.g. studies that were performed in patients not actively receiving treatment at a hospital or hospital outpatient clinic) were discarded, leaving a total of 319 manuscripts. A second, more selective screening was performed to search for specific study inclusion eligibility criteria in the article titles and abstracts. Reviewer 1 (R.R.) screened all the records, while reviewers 2 and 3 (A.N. and T.C.) screened half of the records each.

All the references cited in the initially selected manuscripts were independently screened by the reviewers to reach a consensus; any conflicts were resolved by discussion between the authors. This process yielded a total of 203 articles. Thereafter, full-text screening was performed independently by the three aforementioned reviewers using the same method described above. During this screening process, two consensus meetings were held to ensure a collective understanding of the inclusion and exclusion criteria, thereby reducing the total number of articles to 43.

**Figure 1.**
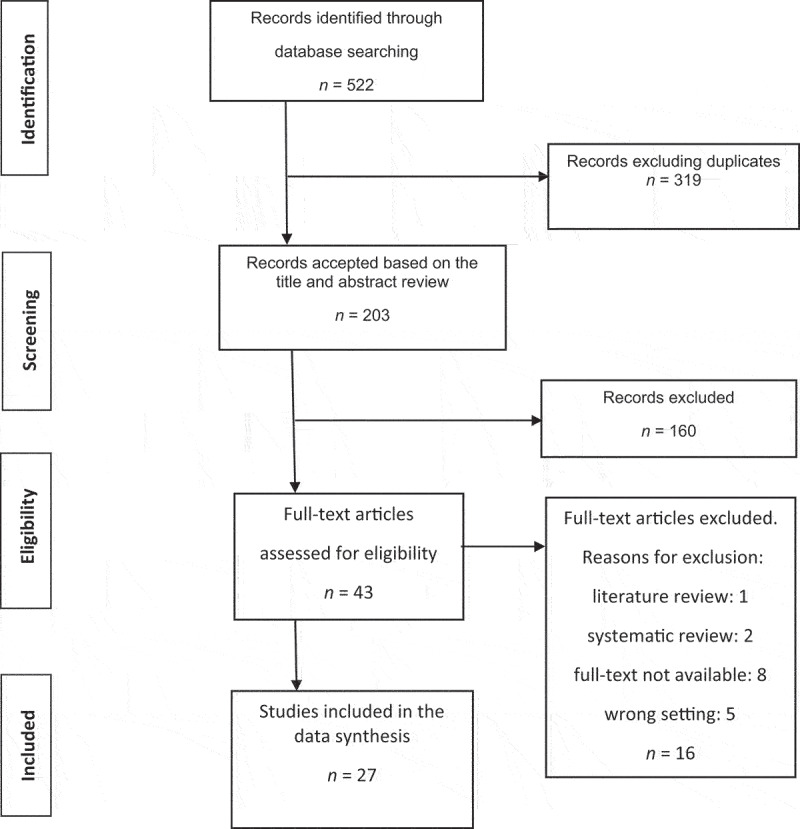
PRISMA flow chart showing the process of manuscript selection for this literature review.

No restrictions were placed on the inclusion of articles in relation to the origin of the studies, sample size, or inclusion of adolescents and young adults with cancer or of articles including the perspectives of parents on MT interventions in young children with cancer. Articles that used MT in patients with other pathologies, completed outside of a hospital setting, including only adults, or had combined MT with other therapies, were excluded. As described in Figure 1, after conducting this literature review selection process, the final sample comprised 27 manuscripts.

#### Stage 4. charting the data

Information was extracted from these 27 selected studies and was tabulated using a data extraction form created by the authors. We carried out a descriptive analysis of each study to help us draw conclusions about the current knowledge regarding MT, as summarised in Tables 2 and 3. The following data categories were extracted from each study: author(s), date, participants, country, cancer type, study design, objectives, primary results, study location, outcome measures, and a description of the MT interventions and methods utilised.

**Table 2.** Summary of the studies used in this scoping review.

Author and Year	Study Design	Objectives	Country	Main Results
Barrera et al., 2002 [24]	Mixed methods	Explore the effectiveness of interactive MT to reduce anxiety and increase the well-being of children and adolescents with cancer.	Canada	There was a significant improvement in the assessments of the patients’ feelings after the use of MT. Parents perceived an improvement in game performance in preschoolers and adolescents, but not in school-age children.
Barry et al., 2010 [32]	Mixed methods	Investigate the effects of MT interventions with the creation of a CD during radiotherapy treatment with paediatric cancer patients.	Australia	The creation of a MTCD was a fun, interesting, and appropriate intervention for children with cancer but there were no significant differences between the intervention and control groups in terms of anxiety and coping strategies used by the patients during radiotherapy treatment.
Bufalini, 2009 [42]	Controlled clinical trial	Examine whether interactive MT can be considered an effective treatment to reduce anxiety in paediatric cancer patients undergoing painful procedures.	Italy	The MT group presented a significant anticipatory anxiety attenuation effect. The degree of satisfaction of children, parents, and staff pointed towards the positive and beneficial role of interactive music during painful procedures.
Burns et al., 2009 [56]	Randomised controlled trial	Investigate the feasibility and preliminary efficacy of a TMV with adolescents and young adults undergoing stem cell transplantation.	USA	The follow-up at 100 days indicated positive trends in patients following TMV interventions in terms of increased hope, spirituality, confidence, and self-transcendence and reduced symptoms of distress and defensive coping.
Burns et al., 2010 [57]	Phenomenology design	Examine parents’ perspectives on the experience of their children undergoing stem cell transplantation while they had participated in a TMV.	USA	Parents perceived that the TMV intervention helped their children by reducing symptoms of distress and allowing them to have a greater sense of control, which allowed their child to better connect with them as well as friends and health workers. Parents also experienced positive emotions.
del Cabral-Gallo et al., 2014 [4]	Quasi-experimental transversal analysis	Assess the efficacy of MT for anxiety management in paediatric cancer patients and their caregivers.	Mexico	The use of MT helped to reduce the anxiety of the caregivers, but there were no significant changes in the children and adolescents with cancer.
Docherty et al., 2013 [61]	Qualitative descriptive design	Describe parents’ perspectives on the utility and significance of a behavioural health MT intervention for AYA with cancer undergoing stem cell transplantation.	USA	Parents indicated that MT intervention helped their children to mitigate physical symptoms, improve their self-esteem, increase acceptance, and to open up their relationships with family and friends. Parents also obtained indirect benefits.
Giordano et al., 2020 [4]	Randomised controlled trial	Evaluate the influence of MT on preoperative anxiety in children with leukaemia undergoing invasive procedures.	Italy	The results supported the potential efficacy of MT in reducing anxiety. More than 90% of the medical staff were very satisfied with the MT interventions.
Haase et al., 2020 [58]	Phenomenology design	Describe, through a randomised controlled clinical trial, the influence the creation of a TMV has on AYA during hospitalisation for stem cell transplantation.	USA	The results reported that creation of a TMV helped patients to overcome distress, explore and identify personal strengths, and enhance their connections with others.
Kemper et al., 2008 [49]	Prospective cohort study	Assess the effect of music in paediatric oncology outpatients.	USA	This work showed that it is possible to evaluate both subjective and objective measures of well-being. There was an improvement in subjective relaxation but there was also an increase in heart rate.
Nguyen et al., 2010 [44]	Randomised controlled trial	Assess whether MT influences pain and anxiety in children undergoing a lumbar puncture.	Vietnam	Decreased pain, heart rate, and respiratory scores in the music group during and after the lumbar puncture. Anxiety and fear were also reduced before and after the procedure.
O’Callaghan et al., 2007 [45]	Qualitative case study	Examine how MT serves as a non-pharmacological anxiolytic for paediatric patients receiving radiotherapy.	Australia	Some patients and their families experienced relief during the stressful wait, others showed improvements on a psychosocial level. Communication between patients and their families was expanded. Fears were expressed metaphorically and there were no adverse experiences.
O’Callaghan et al., 2011 [46]	Constructivist design	Analyse the perspectives of paediatric cancer patients and their parents on the role of music and MT in their children’s lives.	Australia	Children’s adverse cancer experiences are often alleviated by using music. Family, social, and electronic musical interactions promoted children’s resilience and normal development.
O’Callaghan et al., 2012 [27]	Constructivist design	Examine the perspectives of adolescents and young adults on the role of music in their lives.	Australia	MT helped support the phases of cancer treatment and post-treatment by improving self-esteem and social, emotional, and cognitive relationships.
O’Callaghan et al., 2013 [28]	Qualitative design	Examine knowledge of music among patients and the relevance of MT in paediatric cancer patients.	Australia	MT was interpreted as a calming factor that relieved distress, promoted supportive relationships, self-care, playful creativity, and hope.
Polat et al., 2015 [50]	Quasi-experimental design	Examine the effects of MT on anxiety in children with acute lymphoblastic leukaemia undergoing chemotherapy.	Turkey	Anxiety measures were significantly lower in all the patients after the MT intervention in relation to the previous tests.
Robb et al., 2003a [51]	Randomised controlled trial	Examine changes in anxiety and depression levels, according to the phase of bone marrow transplantation, with the use of MT.	USA	The symptoms of depression and anxiety levels varied with the stage of treatment and with the physiological levels of the treatment side effects. Four participants experienced a decrease in anxiety with MT.
Robb et al., 2003b [52]	Randomised controlled trial	Examine the lyrical content of patient-generated songs and compare patient perceptions regarding the effectiveness of a 6-week music experimental condition compared with a no-music condition.	USA	Patients undergoing transplantation who participated in the MT intervention got help to identify and develop their personal strengths in order to deal positively with the stress caused by their disease.
Robb et al., 2008 [26]	Controlled clinical trial	Assess the effectiveness of an AME intervention on three coping-related behaviours (positive facial affect, active engagement, and initiation).	USA	Positive facial effect and active engagement was higher in the group of children with an AME intervention compared with the ML and ASB groups, and the initiation was higher with AME than with ASB.
Robb et al., 2014 [59]	Randomised controlled trial	Examine the efficacy of a TMV intervention performed during the acute phase of patients undergoing stem cell transplantation.	USA	The TMV group reported better coping in the post-intervention and better social and family integration was observed 100 days after the transplant.
Robb et al., 2017 [47]	Randomised controlled trial	Examine the feasibility and acceptability of a AME+P intervention for young children and their parents. Explore changes in child emotional distress and parental emotional distress through an AME+P.	USA	Acceptability was feasible for children but not for parents. Emotional distress was lower for the children in the AME+P group but there were no benefits to parents.
Saghaee-Shahriari et al., 2019 [60]	Quasi-experimental design	Investigate the effectiveness of MT on anxiety sensitivity and self-efficacy in adolescents with leukaemia.	Iran	There was a significant difference in the MT group for anxiety sensitivity and self-efficacy compared to the control group.
Sepúlveda-Vildósola et al., 2014 [53]	Quasi-experimental longitudinal clinical trial	Assess whether MT is effective in reducing the level of anxiety in paediatric cancer patients receiving outpatient chemotherapy.	Mexico	A decrease in anxiety levels was found after the MT intervention.
Tucquet et al., 2014 [54]	Qualitative design	Present the results of a clinical practice review from Australia regarding MT services in hospitals with paediatric cancer patients.	Australia	84% of those surveyed said that MT was a valuable tool, citing distraction from pain and anxiety, psychological improvements, and in self-expression, social relationships, emotional treatment, and better adaptation to hospitalisation.
Uggla et al., 2016 [29]	Randomised controlled trial	Evaluate the previously unexplored effect of MT in children undergoing haematopoietic stem cell transplantation by analysing physiological parameters.	Sweden	Evening heart rates were reduced. There were no significant differences in blood pressure and or oxygen saturation. Stress levels were reduced for 4 to 8 hours, reducing the risk of suffering from post-traumatic stress syndrome.
Uggla et al., 2018 [55]	Randomised controlled trial	Assess the effects of MT during and after haematopoietic stem cell transplantation.	Sweden	The scale showed that the MT group had a higher estimated physical function at the time of discharge but the decrease in pain was not statistically significant. The control group showed better results after the intervention in every domain.
Uggla et al., 2019 [30]	Qualitative design	Explore the experiences of participants and parents regarding the interactive processes during MT interventions.	Sweden	The participant responses showed positive activations of emotions and bonding, distraction from pain and fear, and better interaction, body and sensory expression, trust, self-security, and ability to disconnect.

AME: active music engagement; AME+P: parent-delivered active music engagement; ASB: audio storybooks; AYA: adolescents/young adults; CD: compact disc; ML: music listening; MT: music therapy; TMV: therapeutic music video; music therapy compact disc (MTCD).

**Table 3.** Characteristics of the studies used in this scoping review.

Author and Year	Participants	Type of cancer	Location of the interventions	Outcome Measures		Interventions/Techniques
Barrera et al., 2002 [24]	65 children and adolescents with cancer aged between 6 months and 17 years (6 m–5 y: 33; 6–10 y: 16; 11–17 y: 16).	Leukaemias, brain tumours, osteogenic sarcoma lymphoma, Ewing’s sarcoma, and neuroblastoma.	The Hospital for Sick Children, Toronto, Canada.	FACES PPS PSQ CSQ SSQ The Likert scale was used for the PSQ and SSQ.		MT sessions lasting 45 minutes for 4 to 6 weeks. Music listening, singing, songwriting, improvisation, instrument playing (e.g. bells, drums, pentatonic tone bars, and shakers, among others). No control group.
Barry et al., 2010 [32]	11 children with cancer aged from 6 to 13 years. Outpatient radiotherapy treatment. MT group: 5; standard group: 6.	Brain, kidney, bone soft tissue cancers, and leukaemia.	Peter MacCallum Cancer Centre, Melbourne, Australia.	Three phases: pre-treatment, treatment, and post-treatment. Demographic analysis, paediatric interview (kidcope), and parent and staff questionnaire.		MT waiting room sessions lasting 10–90 minutes and 20–90 minutes in the treatment room. MT group: musical creations through software which were later recorded on a CD. Standard group: standard treatment.
Bufalini, 2009 [42]	39 children with cancer aged 2–12 years and undergoing painful treatment. MT group: 20; control group: 19.	Acute lymphatic leukaemia, non-Hodgkin’s lymphoma, neuroblastoma, osteosarcoma, and medulloblastoma.	Unknown	m-YPAS ICC Scale of emotion, activity, and sociability. Inventory cataloguing the range status in parents with anxiety. Degree of satisfaction of children, parents, and staff with the Barrera questionnaire.		1 MT session. MT group: listening to lullabies, children’s songs, and classical music, among others for 15 minutes. Active music: small percussion instruments, vocals, and body percussion for an unspecified time. Control group: conscious sedation alone.
Burns et al., 2009 [56]	12 participants with cancer aged 11–24 years. Randomised to the music group: 7; randomised to control group: 5; analysed in the music group: 7; analysed in the control group: 2.	Unknown	Unknown	STAIC MOS SDS HHI RSPS IWB Mental Health Scale Child Health Questionnaire. Bodily Pain Scale Child Health Questionnaire. Jalowiec Coping Scale-Revised. Haase Adolescent Resilience in Cancer Scale. Reed Self-Transcendence Scale. Rosenberg Self-esteem Scale. Nowotny Confidence Subscale. LASA Uniscale.		6 MT sessions, lasting 45 to 60 minutes. MT group: created a TMV. Control group: listened to audiobooks.
Burns et al., 2010 [57]	4 mothers and 3 fathers of 6 patients with cancer aged 13–21 years and undergoing a stem cell transplant.	Unknown	In the hospital and/or home of the patient.	Parental interviews were conducted 100 days after the transplantation and lasted between 30 minutes and 90 minutes. Broad range of questions to generate data. Follow-up questions during the interview.		6 MT sessions; created a TMV.
del Cabral-Gallo et al., 2014 [48]	Children and adolescents with cancer aged 6–18 years and undergoing chemotherapy, along with their caregivers. Patients: MT group and control group: 56. Caregivers: MT group and control group: 64.	Unknown	Civil Hospital of Guadalajara Mexico.	Pre and post-intervention measurements. C-MAS-R HAS		MT session lasting a mean of 18 minutes. MT groups: listened to classic, folk, instrumental, and medieval music. Control groups: no music.
Docherty et al., 2013 [61]	16 parents of adolescents and young adults with cancer undergoing stem cell transplantation.	Unknown	Unknown	Semi structured open-ended interview lasting 20 to 60 minutes, performed 100 to 160 days after the transplant. The script included the experiences of the parents regarding participation with their children in the MT.		6 MT sessions; created a TMV.
Giordano et al., 2020 [43]	48 children aged 2–13 years with cancer and undergoing invasive procedures, along with their parents. MT group: 29. Standard group: 19.	Leukaemia	Polyclinic Hospital of Bari, Italy.	m-YPAS, in the waiting room on the day of the procedure and in the operating room. Questionnaire edited by Zanchi and Acler for the medical staff		6 MT sessions lasting 15 to 20 minutes. Method taken from the Free Improvisation Therapy model. MT group: instrument playing, improvisation, singing, and music listening. Standard group: standard treatment.
Haase et al., 2020 [58]	14 cancer patients aged 13–22 years and undergoing stem cell transplantation.	Unknown	Unknown	Semi-structured interviews lasting 4 to 24 minutes. Broad initial question to generate data. Questions for deeper reflections asked during the interview.		MT group: created a TMV. Control group: listened to audiobooks.
Kemper et al., 2008 [49]	63 children and adolescents (ambulatory) with cancer aged up to 17 years.	Acute lymphoblastic leukaemia and myelogenous leukaemia.	Brenner Children’s Hospital, Winston- Salem, North Carolina, USA.	In both visits the parents completed the VAS before and after the MT. Patients’ heart rates were monitored during the treatments to calculate their HRV.		Visit 1: patients rested for 20 minutes. Visit 2: listening to Heart Zones music by Doc Childre for 20 minutes. No control group.
Nguyen et al., 2010 [44]	40 children with cancer aged 7–12 years and subjected to a lumbar puncture. Music group: 20; control group: 20.	Leukaemia	National Paediatric Hospital, Hanoi, Vietnam	STAI, scores before and after the procedure. NRS, HR, BP, RR, and SpO_2_ were recorded throughout the procedure with the children listening or not listening to music according to their groups. Interview with three open questions.		1 MT session lasting an average of 23 minutes. Music group: music listening with headphones (children’s and Vietnamese songs). Control group: headphones without music.
O’Callaghan et al., 2007 [45]	39 outpatient children with cancer aged up to 14 years and receiving radiotherapy, along with 63 families and friends.	Brain tumours, sarcomas, neuroblastoma, and leukaemia.	Peter MacCallum Cancer Centre, Melbourne, Victoria, Australia.	Individual case reports.		85 MT sessions lasting an average of 30 minutes. Sessions while waiting and during the treatment. Instrument playing (e.g. synthesiser, autoharp, guitar, and percussion instruments). Songwriting, singing, and improvisation, among others.
O’Callaghan et al., 2011 [46]	26 children with cancer aged up to 14 years along with their parents.	Leukaemias, lymphomas, neuronal and non-neuronal solid tumours.	Three hospitals in Melbourne, Victoria, Australia.	Semi-structured interviews lasting an average of 16 minutes were conducted with 26 patients and 28 parents.		Singing, instrument playing, music listening, and DVD creation, among others.
O’Callaghan et al., 2012 [27]	12 cancer patients aged 12–25 years.	Sarcomas, solid tumours, leukaemia, melanoma, pineal germinoma, and metastatic disease.	Peter MacCallum Cancer Centre, Melbourne, Victoria, Australia.	Semi-structured interviews lasting an average of 57 minutes were conducted.		Music listening, singing, songwriting, dancing, and instrument playing, among others.
O’Callaghan et al., 2013 [28]	32 children and adolescents with cancer aged 2–18 years.	Unknown	Three Hospitals in Melbourne, Victoria, Australia.		Four music therapists that had worked with the patients were interviewed.	Music listening, songwriting, singing, and instrument playing, among others.
Polat et al., 2015 [50]	28 children and adolescents with cancer aged 5–15 years and undergoing chemotherapy. Pre-test and post-test single-group design.	Acute lymphoblastic leukaemia.	Turkey University Hospital.		VAS at the beginning and end of the intervention. Questions for mothers and children.	MT sessions lasting 15 to 30 minutes. MT group: listening to songs from The Four Seasons. No control group.
Robb et al., 2003a [51]	6 children and adolescents with cancer aged 9–17 years and undergoing bone marrow transplantation. Music group: 3. Group without music: 3.	Peripheral T-cell lymphoma, desmoplastic small cell tumour, acute lymphocytic leukaemia, Ewing’s sarcoma, and non-Hodgkin’s lymphoma.	Children’s Mercy Hospital, Kansas City Missouri, USA.		STAIC CDI	Contextual Support Model of MT (Robb). 6 MT sessions. Music group: created a TMV. Group without music: preferred activity of the patients.
Robb et al, 2003b [52]	6 children and adolescents with cancer aged 9–17 years and undergoing bone marrow transplantation. Music group: 3. Group without music: 3.	Peripheral T-cell lymphoma, desmoplastic small cell tumour, acute lymphocytic leukaemia, Ewing’s sarcoma, non-Hodgkin’s lymphoma, and acute myelogenous leukaemia.	Children’s Mercy Hospital, Kansas City Missouri, USA.		STAIC CDI	Contextual Support Model of MT (Robb). 6 MT sessions. Music group: created a TMV. Group without music: preferred activity of the patients.
Robb et al., 2008 [26]	83 children with cancer aged 4–7 years. Experimental group (AME): 27; control group (LM): 28; control group (ABS): 28.	Unknown	Mercy Hospitals and Clinics, MO. State Milton Medical Centre S. Hershey, Hershey, PA. Rainbow Hospital for Babies and Children Cleveland, OH. Children’s Hospital University of Iowa, Iowa City, IA. Riley Hospital for Children, Indianapolis, IN.		Average frequency estimates were calculated for three behaviours related to coping: positive facial effect, active commitment, and initiation.	1 MT session lasting 30 minutes. Experimental group (AME): instrument playing, and singing, among others. Control group (LM): music listening for children. Control group (ABS): 2 picture books for children with audio narration.
Robb et al., 2014 [59]	113 patients aged 11–24 years with cancer undergoing a stem cell transplantation. Music group: 59; control group: 54.	Leukaemia, lymphoma, and solid tumours.	Riley Children’s Hospital and Indiana University Hospital Indianapolis, IN. Children’s Mercy Hospitals and Clinics Kansas City, MO. Children’s Healthcare of Atlanta/Emory University Atlanta, GA. Methodist Children’s Hospital and Texas Transplant Institute of San Antonio, TX. St. Louis Children’s Hospital and Barnes-Jewish.		Intervention and 100 days post-transplantation. RIM McCorkle Distress Symptom Scale. Mishel Uncertainty in Illness Scale. Jalowiec Coping Scale-Revised. Reed Spiritual Perspective Scale. Perceived Social Support-Health Care Providers. Perceived Social Support-Friends. Perceived Social Support-Family. Family Adaptability/Cohesion Scale. Parent-Adolescent Communication Scale.	6 MT sessions over three weeks. Contextual Support Model of MT (Robb). MT Group: created a TMV. Control group (ABS): listened to the patient’s choice of audiobook from among 15 options.
			Hospital St. Louis, MO. Duke Children’s Hospital, Durham, NC. Helen DeVos Children’s Hospital, Grand Rapids, MI. C.S. Mott Children’s Hospital, Ann Arbor, MI.		Family Strengths Scale. Herth Hope Index. Reed Self-Transcendence Scale. Haase Resilience in Illness Scale.	
Robb et al., 2017 [47]	16 children with cancer aged 3–8 years and 12 parents. AME+P group: 9; ABS control group: 7.	Leukaemia and tumours.	Riley Hospital for Children, Indiana, USA.		AME Parent Delivery Checklist. Positive Facial Affect. Child Engagement. Facial Affect. POM-SF IES-R Scores for positive side effects and active engagement. Interviews with parents to evaluate the AME+P 30 days after the intervention.	3 MT sessions lasting 45 minutes (AME+P) or 35 minutes (attention control). Contextual Support Model of MT (Robb). AME+P group: music play kit. ABS control group: listened to audiobooks.
Saghaee-Shahriari et al., 2019 [60]	30 adolescents with cancer. Ages not specified. MT group: 15; control group: 15.	Leukaemias	Health centres in Tehran, Iran.		ASI General Self-Efficacy Scale.	14 MT sessions lasting 90 minutes.
Sepúlveda-Vildósola et al., 2014 [53]	22 children and adolescents with cancer aged: 8–16 years.	Non-Hodgkin’s lymphoma, acute lymphoblastic leukaemia, and acute myeloid leukaemia.	Paediatric Hospital at the XXI Century National Medical Centre, Mexican Social Security Institute.		Visual analogue numerical scale, first without MT and later with the MT.	MT session involving listening to the music of J. Thompson, lasting an average of 20 minutes for 2 months.
		Hodgkin’s disease, tonsillar lymphoma, osteosarcoma, histiocytosis, primitive neuroectodermal tumour, and rhabdomyosarcoma.	Mexico			
Uggla et al., 2016 [29]	24 children and adolescents with cancer aged up to 16 years and undergoing stem cell transplantation. MT group: 13 (1 dropout); control group: 11 (2 dropouts).	Acute myeloid leukaemia, acute lymphatic leukaemia, myelodysplastic syndrome, and non-malignant	University Hospital Karolinska-Huddinge, Stockholm, Sweden.		Measurements of the heart rate, blood pressure, and oxygen saturation; normal scanning protocols between 7 and 8 in the morning and 6 and 8 in the afternoon.	MT sessions lasting 45 minutes twice a week for a mean of 4 to 6 weeks. The MT method originated from two models: the Nordoff–Robbins Creative MT and Juliette Alvin’s Free Improvisation Therapy. MT group: singing, instrument playing, and music listening. Control group: standard treatment.
Uggla et al., 2018 [55]	29 children and adolescents with cancer aged from 2 months to 17 years and undergoing a stem cell transplantation. MT group: 14; control group: 15.	Acute myeloid leukaemia, acute lymphatic leukaemia, myelodysplastic syndrome, and non- malignant	University Hospital Karolinska-Huddinge, Stockholm, Sweden.		Paediatric quality of life inventory 4.0 (generic basic scale PedsQL 4.0). Inventory 3.0 cancer module (PedsQL 3.0 cancer module). The research nurse subjectively documented the patient mood on a five-point scale. Three-point Likert scale.	MT sessions lasting 45 minutes twice a week for an average of 4 to 6 weeks. MT group: singing, instrument playing, and music listening. Children aged under 18 months interacted with their parents, commitment based on body language.
					Five-point Likert scale. Astrid Lindgren Pain Scale. Visual Analogue Pain Scale. Lansky Gaming Performance Scale.	Control group: standard treatment.
Uggla et al., 2019 [30]	6 children and adolescents with cancer aged 1–18 years and undergoing a stem cell transplant, along with 6 family groups.	Unknown	University Hospital Karolinska-Huddinge, Stockholm, Sweden.		Collaborative research MT interview and the child-parent interview, 7–13 months after the transplantation lasting 45–60 minutes.	Singing, improvisation, instrument playing, and songwriting, among others.

AME: active music engagement; AME+P: parent-delivered active music engagement; ASB: audio storybooks; ASI: Anxiety Sensitivity Index; BP: blood pressure; CD: compact disc; CDI: Children’s Depression Inventory; C-MAS-R: Manifest Anxiety Scale in Children-Revised; CSQ: questionnaires to children; DVDs: digital versatile disc; FACES: Faces Pain Scale; HAS: Hamilton Anxiety Scale; HHI: Herth Hope Index; HR: heart rate; HRV, heart rate variability; ICC: Induction Completion List; IES-R: Impact of Events Scale-Revised; IWB: Index of Well-Being; ML: music listening; MOS: Short-Form Health Survey-Medical Outcomes Study; MT: music therapy; m-YPAS: Modified Yale Pre-operative Anxiety Scale; NRS: Numeric Rating Scale; POMS-SF: Profile of Mood States-Short Form; PPS: Play-Performance Scale; PSQ: Satisfaction Questionnaires Completed by Parents; RIM: Disease Resistance Model; RR: respiratory rate; RSPS: Reed Spiritual Perspective Scale; SDS: McCorkle Symptom Distress Scale; SpO_2:_ oxygen saturation; SSQ: questionnaires to staff; STAI: Spielberger State-Trait Anxiety Inventory; STAIC: State-Trait Anxiety Inventory for Children; TMV: therapeutic music video; VAS: Visual Analog Scale.

## Results

### Characteristics of the publications

Of the 27 publications that met the inclusion criteria, 16 (59.25%) were quantitative studies, nine (33.33%) were qualitative studies, and two (7.40%) were mixed studies. Ten of them were conducted in the USA (37.03%), six in Australia (22.22%), three in Sweden (11.11%), two in Mexico (7.40%), two in Italy (7.40%), and one each in Iran, Canada, Turkey, and Vietnam. Tables 2 and 3 provide more detailed information on all the publications included in this review.

The most common benefits found in the articles considered were psychological (*n* = 18), social well-being (*n* = 11), emotional (*n* = 8), physical (*n* = 8), self-esteem (*n* = 7), cognitive (*n* = 7), and physiological (*n* = 4). The research had included a broad range of patient ages, with most being children and adolescents aged between 2 months and 18 years [24,28–30,42–49]. However, in some studies, the age range was 2–14 years [26,32,50–55] and in others it was between 11 and 25 years [27,56–59].

A total of 18 studies mentioned the type of cancer affecting their participants. The most frequent were unspecified leukaemias (*n* = 9), acute lymphocytic leukaemia (*n* = 8), lymphomas (*n* = 7), brain tumours (*n* = 5), acute myelogenous leukaemia (*n* = 4), osteogenic sarcoma (*n* = 3), Ewing’s sarcoma (*n* = 3), neuroblastoma (*n* = 3), non-Hodgkin’s lymphoma (*n* = 3), non-solid neuronal tumours (*n* = 3), and other less frequent neoplasms. Tables 2 and 3 provide more detailed information on the publications included in this review; Table 3 shows the heterogeneity of the outcome measures (in the form of questionnaires, scales, interviews, and physiological measures, among others).

### The effect of music therapy in children and adolescents with cancer

Most of the results had demonstrated the effectiveness of MT in the reduction of anxiety in childhood cancer patients undergoing painful procedures, chemotherapy, or radiotherapy during their hospital stay [46,47,49–52,60]. The study by del Cabral-Gallo et al. [44] showed that after an MT intervention, anxiety was also reduced among caregivers, although the results in children and adolescents did not show significant effects in this study. According to the authors, this may have been because the physical experience of the disease can influence the emotional responses of patients, which, in turn, can affect their anxiety levels. Similarly, Nguyen et al. [52] also found a significant reduction in pain in their patients after MT. Finally, Barrera et al. [24] demonstrated that patients expressed their feelings better following MT, thus reducing parental anxiety because of better interaction and communication.

Several studies examined the effects of MT sessions in children and adolescents with cancer in different phases of stem cell transplantation. One showed that there tended to be an improvement in the ability of these patients to cope, trust, self-transcend, and hope after MT [56]. Similarly, two studies reported that MT can offer patients a means to overcome distress, identify personal strengths, and improve relationships with others [48,58]. In turn, Robb et al. [59] showed that patients in the MT intervention group coped better during the acute phase of stem cell transplantation. The work by Uggla et al. [43] showed that patients who received MT sessions had greater physical function and improved mood at the time of discharge. Lastly, another study revealed that patient responses to MT interventions led to more positive emotions and improved interactions with others, thereby allowing them to better deflect the fears and concerns arising during the treatment process [30].

Other work looked at the capacity of patients to self-regulate in order to deal with stress in the hospital environment. Thus, Robb et al. [26] found that children in their active musical engagement (AME) group had more positive facial expressions and more often and more actively participated than those who listened to music or audiobooks in their ML and ASB groups, respectively. In this work, the essential elements of intervention in the AME group were: (1) music-based activities; (2) providing children with the opportunity to choose materials and using live music to support their autonomy; and (3) interventions guided by music and certified for that purpose. Similarly, another study examined the viability and acceptability of AME with the musical activities being delivered to their children by their parents (AME+P). They explored the anguish of both the patients and their parents and found a significant improvement in the levels of distress and coping abilities in the patients but not the parents [55].

Finally, regarding the evaluation of physiological parameters, Uggla et al. [29] showed that MT helped reduce the heart rate of patients, while Nguyen et al. [52] found a decrease in both their heart and respiratory rates. In contrast, research by Kemper et al. [45] showed an increase in heart rate after the delivery of MT, which, according to the authors, could be related to the choice of songs used for these sessions.

### Perceptions about the music therapy interventions

The study by O’Callaghan et al. [27] examined the points of view of adolescents with cancer on the role of music in their lives. The results demonstrated how MT helped their psychological well-being and improved their self-esteem and social relationships, among other parameters. Another study revealed that adverse cancer experiences in children are often alleviated by MT and that interactions between patients and family members are enhanced by using MT, thereby favouring patient resilience [54]. Furthermore, two other studies described the opinions of parents on the usefulness of MT interventions with their children, indicating that they believed that MT had helped their children physically, emotionally, and socially.

Moreover, MT also led to indirect benefits among the parents, including disconnection from their situation and improvements in their mood [57,61]. The study by Tucquet and Leung [42] described the ability of MT to facilitate family relationships, emotional expression, and self-expression, among other psychological benefits. In turn, O’Callaghan et al. [28] examined the relevance of MT in children and adolescents with cancer from the perspective of music therapists. They highlighted the fact that, in the opinion of the music therapists, their interventions could alleviate anxiety, promote supportive relationships, self-care, creativity, and hope in their patients. Finally, Barry et al. [32] demonstrated that MT provided a happy and positive experience for healthcare personnel. Similarly, Giordano et al. [51] found that physicians positively assessed the use of MT in children and adolescents with oncological pathologies.

### Characteristics of the music therapy sessions

The 27 publications used in this scoping review included information on the MT services or musical interventions used. MT sessions were offered either individually (*n* = 18) or both individually and in groups (*n* = 9). Regarding the methodology used, nine studies used active MT methods (*n* = 9), six employed receptive MT methods (*n* = 6), and 13 utilised a mix of both these MT methods (*n* = 13). Other methodologies used were therapeutic music videos (TMV), creation techniques [56–59,61], song composition and/or digital video production techniques [47,48], improvisation, instrument playing [45], creation of an MT compact disc (MTCD) [32], singing, dancing, listening to music, and improvisation, among others [24,26–30,42,43,50,51,54,55].

The length of the MT sessions varied between 15 and 90 minutes. The number of MT sessions also varied from one (*n* = 4), three (*n* = 1), six (*n* = 5), 14 (*n* = 1), and up to 85 sessions (*n* = 1). Both these aspects, i.e., the number of MT sessions and their length, were cited in 13 publications while other publications cited only the number of MT sessions (*n* = 4) or their duration (*n* = 3). In a total of 5 publications, neither the number of MT sessions nor their duration was cited. In terms of analysis, several articles detailed the theoretical model upon which their MT interventions had been based, such as the Contextual Support Model of Music Therapy [26,47,48,55,59], both the Nordoff–Robbins Creative Music Therapy model and Juliette Alvin’s Free Improvisation Therapy model [29], or the latter model alone [51].

Some publications specified the type of music used in the musical interventions, including lullabies, folk songs, children’s songs, pop and classical music [50], music by J. Thompson [49], classical, folk, instrumental, and medieval music [44], children’s songs and Vietnamese songs [52], songs from The Four Seasons [46], or some of Doc Childre’s Heart Zones songs [45]. Research by Robb et al. [26] listed the song titles used in their MT interventions: the opening song to *Willoughby Wallaby Woo*, the action songs *Five Little Monkeys* and *Five Little Speckled Frogs*, versions of the songs *I am a Great Musician* and *Momma Don’t Allow*, illustrated storybook songs *Wheels on the Bus* and *Down by the Bay*, and the closing song *Time to Say-Bye*. Other publications listed the musical instruments used as percussion, classical guitar, omnichord, and keyboard [24], guitar, keyboard, percussion instruments, autoharp, and omnichord [53], and percussion instruments, vocal, and body percussion [50]. Table 3 provides more detailed information about all these variables.

## Discussion

The changes that children and adolescents with cancer undergo influence their moods, and the quality of their lives [3,4]. However, the use of MT can be of immense help in coping with the disease processes of these patients, leading us to conduct this present scoping review. Our results add to the findings of other studies that have already confirmed the feasibility and efficacy of the use of MT in children and adolescents with cancer. We observed that, despite the heterogeneity of the methodological designs of the studies we considered, many of them had the same common goals. These aims included examining the viability and efficacy of MT as well as investigating the perspectives of patients and parents on the relevance of MT.

As indicated by previous systematic reviews [33,34], our review showed that, due to the wide range of methodological designs used, and the heterogeneity of the MT sessions employed and patient ages, etc., it is not easy to reach general, overall conclusions about the effect of MT. However, several studies have shown that MT can significantly reduce anxiety [46,47,49–52,60] in paediatric cancer patients. For example, the study by Giordano et al. [51] indicated that reducing preoperative anxiety with MT helps children with cancer to go to the operating room with less fear. Regarding anxiety, although the objectives addressed in children and adolescents with cancer differed from one study to another, the results reported in this review were consistent with those conducted by other systematic reviews in cancer patients of all ages [62–64]. These previous reviews evaluated anxiety during painful procedures, when administering chemotherapy, during the hospital stay, and preoperatively and found that the musical interventions had had beneficial effects.

We also found a few studies that evaluated how MT influenced physiological parameters. For example, Kemper et al. [45] showed an increased heart rate was associated with the MT sessions in children and adolescents with cancer. On the contrary, the study by Uggla et al. [29], which was also conducted in children and adolescents with cancer, showed that there were no significant differences in blood pressure or oxygen saturation, but there was a reduction in the nocturnal heart rate of these patients. Finally, Nguyen et al. [52] performed a study in children with cancer and showed that both their respiratory rates and heart rates were lower after MT.

It is also interesting to discuss the effects of MT on the reduction of pain in children and adolescents with cancer; three studies found no significant pain reduction [24,43,56], while another [52] did find such a reduction. Studies completed in other patient profiles, such as adult oncology patients [17,65], burn patients [66], and palliative care patients [67], have also shown significant results in reducing pain with MT. Therefore, we suggest that future lines of research should be implemented to evaluate the pain parameters in the paediatric population.

Regarding the perceptions of patients and their families, five studies showed that MT interventions were beneficial in terms of improving patient psychological, physical, emotional, and social well-being, and so on. Moreover, they described how parents also obtained indirect benefits from MT sessions [27,42,54,57,61]. Furthermore, the study by Robb et al. [59] showed that patients reported improvements in their social support and family cohesion. Other work has shown that the interactions between patients and their parents during MT sessions help patients to experience self-knowledge, improve their self-regulation, and face and manage the treatment period in hospital [30], thereby improving their quality of life and reducing their isolation during the disease processes [68]. Specifically, O’Callaghan et al. [28] reported that music therapists had indicated that the children and adolescents with cancer in their MT sessions should have access to music suiting their preferences. They also demonstrated that MT could help these patients by promoting supportive relationships, playful creativity, emotional expression, self-care, and adversity management, thereby positively influencing their health.

Two studies showed the MT sessions also had a very positive effect on health personnel [32,51] and suggested that future research focus on assessing the participation of health personnel in such sessions. In addition, some studies reported very positive results regarding MT interactions between children and adolescents with cancer and their families [28,53–55,61]. Interactions between parents and children are considered important to help offer relief to patients in adverse situations and to create a greater connection between them [54]. In addition, parent–child participation in MT sessions can be useful for regulating children’s emotions and pain management, leading to improvements in their social interactions and enhancing their confidence and body expression [29].

The music was chosen by the patients in most of the studies in which receptive or mixed MT methods had been used in the MT sessions [27–30,43,46,52,54]. One study in which the researchers had selected the music [49] obtained similar results, while two others did not produce the expected results [44,45]. This may suggest that patients should have access to their preferences for the musical content and preferred instruments during MT interventions [26–28]. Most of the active MT methods used percussion, guitar, and keyboard instruments [24,53], as well as in the creation of TMVs. These tools were well accepted by children and adolescents with cancer [56–59]. The studies we considered all recommended future research to design and standardise the protocols used for clinical practice and to unify the intervention criteria at the national and international levels [42].

## Limitations

The present review had some limitations. First, only articles published in English and in Spanish were included. Second the results could not be generalised because of the heterogeneity of the studies included. Nonetheless, many of them had common objectives, such as examining the viability and efficacy of MT. Thirdly, the studies considered had included a wide range of patient ages, clinical disease stages, and end measures, as well as MT methods and session procedures and techniques. Thus, these factors may have limited our ability to unify the criteria for the MT intervention results. However, given the characteristics of this study, this work provides a solid basis for the use of MT with children and adolescents with cancer and can serve as a guide in clinical practice and for future research.

## Conclusions

This scoping review showed that MT interventions are well received not only by children and adolescents with cancer but also by their families, music therapists, and health professionals. It also demonstrated that the use of MT with children and adolescents with cancer is a viable and effective option to improve their quality of life. Given the heterogeneity of the studies considered in this current work, this review also demonstrated the need to continue research in this field. This will allow therapists to offer the benefits of MT based on solid scientific evidence as applied to children and adolescents with cancer.

## Paper context

Several studies have evaluated MT interventions, but very few comprehensive reviews have been conducted in this field to date. This article reviews the available literature and summarises the results. MT is an effective intervention that reduces paediatric cancer patients and family anxiety and stress. MT is well received and appreciated by this population and their health care clinicians. This article serves as a practical clinical guide to MT and demonstrates where the need for continued research in this area should be directed.
